# First Results of the Implementation of the Doppler Backscattering Diagnostic for the Investigation of the Transition to H-Mode in the Spherical Tokamak Globus-M2

**DOI:** 10.3390/s23020830

**Published:** 2023-01-11

**Authors:** Anna Ponomarenko, Alexander Yashin, Gleb Kurskiev, Vladimir Minaev, Alexander Petrov, Yuri Petrov, Nikolay Sakharov, Nikita Zhiltsov

**Affiliations:** 1 Plasma Physics Department, Peter the Great St. Petersburg Polytechnic University, 195251 St. Petersburg, Russia; 2 Plasma Research Laboratory, Ioffe Institute, 195251 St. Petersburg, Russia

**Keywords:** tokamak, plasma diagnostics, H-mode, Doppler backscattering, plasma turbulence

## Abstract

This paper presents the first results of a study of the LH transition on the new spherical Globus-M2 tokamak using the Doppler backscattering (DBS) diagnostic. New data characterizing the H-mode of discharges with higher values of the plasma parameters, such as magnetic field B_t_ up to 0.9 T and plasma current I_p_ up to 450 kA, were collected and analyzed. An upgraded neutral beam injection (NBI) system was used to initiate the LH transition. DBS allows the measurement of the poloidal rotation velocity and the turbulence amplitude of the plasma. The multi-frequency DBS system installed on Globus-M2 can simultaneously collect data in different areas spanning from the separatrix to the plasma core. This allowed for the radial profiles of the rotation velocity and electric field to be calculated before and after the LH transition. In addition, the values and temporal evolution of the velocity shear were obtained. The associated turbulence suppression after the transition to the H-mode was investigated using DBS.

## 1. Introduction

Developing an accurate and reliable diagnostic system for the successful study of the various phenomena taking place in a hot plasma of a magnetic confinement device, such as a tokamak, is a task of utmost importance. For this reason, a wide range of methods and techniques have been proposed and tested on different machines over the years [1]. The effective implementation of any diagnostic is a priority for any experimentalist, as their results will have a great influence on the capabilities of any future fusion device.

An example of the successful implementation of the diagnostics of Doppler backscattering (DBS) for the investigation of plasma processes during the transition to H-mode is presented in this paper. The H-mode is an intriguing and illustrative phenomenon to discuss. The results obtained and analyzed can highlight the usefulness and showcase the range of data that is possible to obtain using such a diagnostic system. The transition to the H-mode has been the topic of a multitude of studies throughout the years on various devices all over the world because of its significant importance and interest to the plasma physics community. Ever since its discovery [2], a lot of effort has been made to reproduce it in all major toroidal confinement devices and discover its properties along with the main causes of the transition to this high confinement regime. A lot of progress has been made in understanding the H-mode, and it is now proposed that it will be the operational mode for future fusion devices, such as the International Thermonuclear Experimental Reactor (ITER) [3]. The H-mode is an enhanced confinement regime characterized by an increase in energy confinement time. A large variety of methods are used to study the phenomena that accompany the LH transition [4]. Much of the experimental work conducted indicates that the H-mode seems to be defined by the anomalous transport in plasma. It was shown that the suppression of such turbulence perturbations leads to the transition to improved confinement and it was also recognized that the E × B shear plays a key role in this process [5,6]. However, the mechanisms responsible for the formation of this sheared rotation vary drastically and remain somewhat unclear. This is why detailed theoretical work and various simulations alongside experimental research are aimed at gaining insight into the mechanisms responsible for this operational mode. 

It is important to point out, as it was also noticed, there is a distinct difference between the improved confinement in spherical tokamaks (ST; characterized by a smaller aspect ratio) and that witnessed in other types of devices. This makes the topic a subject worth investigating. One key observed difference between the devices Mega Ampere Spherical Tokamak (MAST), National Spherical Torus Experiment (NSTX), and Globus-M/M2 is the fact that the energy confinement time has a strong dependence on the values of the toroidal magnetic field, which has not previously been observed [7,8,9]. It is worthy of note that all of the aforementioned tokamaks differ in parameters and yet still show a similar dependence on the magnetic field, which contradicts the conventional τ_E,98y,2_ scaling [8]. It was also determined that there seems to be a strong correlation between the increase in energy confinement time and the decrease in collisionality, which is seen as an indication of favorable plasma parameters for the future [10]. All this leads to the question of what other properties of the H-mode or the LH transition deviate in spherical tokamaks in comparison to more conventional devices. 

The results of experimental research on the transition to the H-mode on the spherical Globus-M2 tokamak using the DBS method are presented in this paper, which is structured as follows. The next section is devoted to the description of the DBS method and its basic principles. This includes a description of the multi-frequency DBS system installed on the Globus-M2 and used in the experiments. After that, the LH transition on Globus-M2 is discussed. Along with that, the DBS measurements of the velocity and turbulence behavior are analyzed, as the LH transition is thoroughly investigated. Finally, a summary of all the results is presented.

## 2. Doppler Backscattering

**Figure 1:**
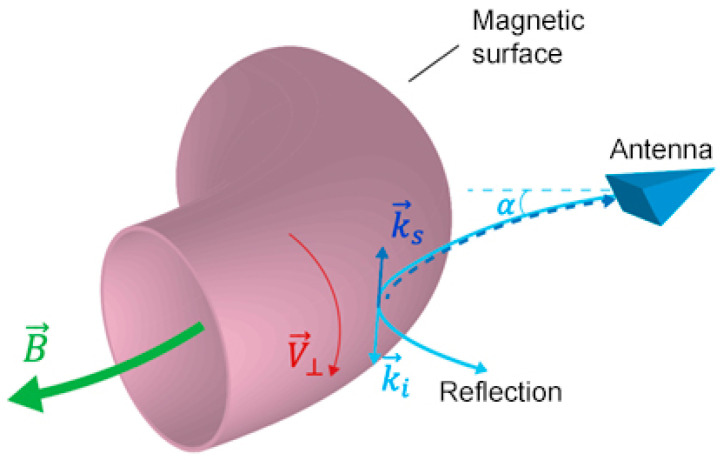
Schematic of a microwave beam incident on the reflection layer.

Doppler backscattering (DBS, also referred to as Doppler reflectometry) is a diagnostic method that is based on the probing of the plasma using microwave beams at oblique incidence [11,12]. In the case of the existence of a cut-off layer for the probing beam (i.e., the point that limits the trajectory of the probing beam), one can detect backscattering of microwave radiation, which takes place predominantly near the cut-off (see the schematic representation in Figure 1). This means that this diagnostic allows for local measurements of the plasma parameters. 

Analysis of the reflected probing beam signals leads to the observation that the Doppler shift in the backscattered frequency spectra is directly proportional to the perpendicular rotation velocity of the density turbulence moving with the plasma:

(1) $$\omega_{Doppler}=\;\vec{V}\cdot \vec{k}\;=V_{⏊}k_{⏊}$$

This velocity component $V_{⏊}$ corresponds to a rotation in the direction of the diamagnetic or the E × B drift (see Figure 1). Due to this, the radial electric field can also be calculated using DBS measurements. To construct a velocity or electric field profile, separate measurements of the cut-off layer positions of different probing beam frequencies are required. This would allow following the inhomogeneity of the velocity. This means the velocity shear can be calculated using DBS, which is one of the key parameters that influence the transition to the H-mode. $k_{⏊}$ is the wave vector of scattering fluctuations and its size can be determined by Bragg’s law:

(2) $$\vec{k_{⏊}}=\vec{k_{i}}-\vec{k_{s}}$$

where $\vec{k_{i}}$ is the wave vector of the incidence wave, and $\vec{k_{s}}$ is the wave vector of the scattered wave of electromagnetic radiation. These vectors are shown in Figure 1.

In order to obtain the Doppler frequency shift, the Doppler backscattering method records two signals, the in-phase (I) and quadrature (Q) signals, which are then formed into a complex IQ signal. Then, the method calculates the derivative of the phase of the complex signal (time dependence of the frequency shift of the scattered signal) or the time dependence of the shift of the spectra of short parts of the IQ signal [12]. Both approaches exhibit approximately the same temporal behavior and are thus deemed equally appropriate for use.

Apart from that, the amplitude of the complex IQ signal directly corresponds to the plasma turbulence behavior. It determines the backscattered power in a given wave number region near the cut-off. This means using DBS it is possible to investigate the properties of turbulence during the LH transition and in the H-mode. 

This method has been used on a variety of tokamaks and has garnered results regarding the rotation velocity and radial electric field of the plasma [12,13,14,15,16,17,18,19]. On Globus-M, this diagnostic has also been successfully utilized but only for the study of plasma oscillatory processes, not to investigate the transition to H-mode [20]. Moreover, a multitude of other phenomena was discovered and fruitfully studied, such as geodesic acoustic modes (GAMs), limit-cycle oscillations (LCOs), Alfven eigenmodes (AEs), filaments, and others [21,22,23,24]. As a result, it was deemed appropriate to continue research on the LH transition and the H-mode using the DBS method on the new and upgraded Globus-M2 tokamak.

### 2.1. Doppler Backscattering to Study the LH Transition and the H-Mode

**Figure 2:**
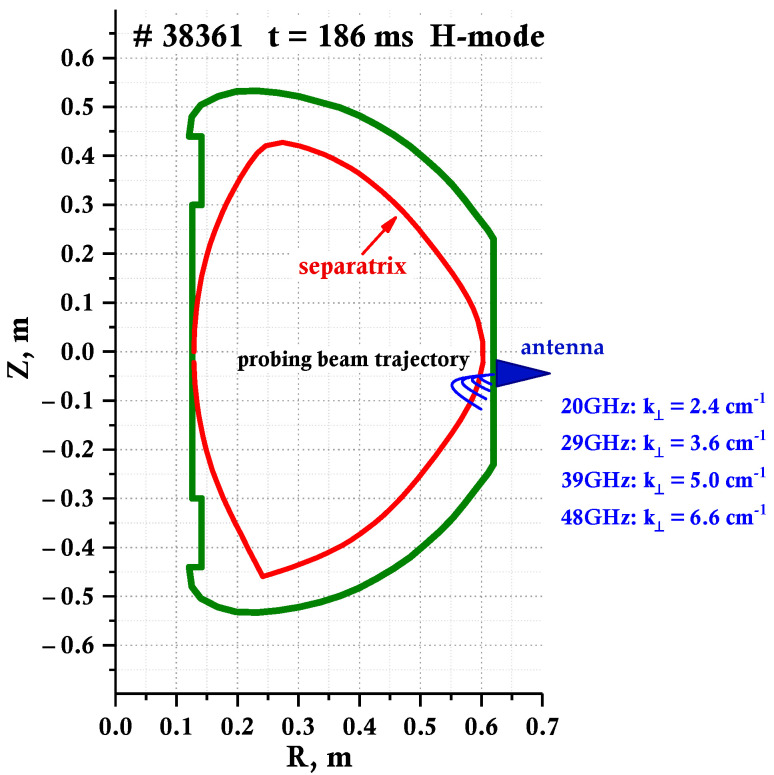
Ray tracing for the four-frequency DBS system used to study the LH transition.

On the Globus-M2 tokamak, the installed DBS system that uses dual-frequency probing investigated the LH transition [25]. Two pairs of fixed frequencies were chosen: 20, 29 GHz and 39, 48 GHz. Each frequency channel includes a microwave circuit with dual homodyne detection, which allows for the quadrature detection of backscattered radiation. Two steerable antennas are used to probe the plasma with O-mode microwaves. One of the antennas is used for frequencies of 20 and 29 GHz, while the second one is for 39 and 48 GHz frequencies. The antennas can be rotated in both the poloidal and toroidal directions. They are also connected to stationary DBS equipment using flexible waveguides. In initial experiments, the DBS system was positioned in the same way as on the Globus-M. The system was located in the equatorial plane of the tokamak. An example of ray tracing performed for the four DBS channels, calculated using a special ray tracing code, written in the Wentzel–Kramers–Brillouin (WKB) approximation and for the Globus-M2 geometry, is shown in Figure 2 [26]. The code requires EFIT data about the magnetic field configuration and density measurements using Thomson scattering diagnostics to allow for an accurate calculation of the probing beam trajectory. Figure 2 depicts the poloidal cross-section of the Globus-M2 tokamak with a series of blue lines indicating the trajectory of the probing beams that correspond to the four probing frequencies available: 20, 29, 39, and 48 GHz. The detection region covered a considerable interval of normalized small radii ρ = 0.7–1.1 for typical discharges, discussed in the following section.

## 3. LH Transition and H-Mode on Globus-M2

In experiments on the Globus-M2 tokamak (major radius R ≈ 0.35 m, minor radius a ≈ 0.22 m), a two-fold increase in the toroidal magnetic field (compared to the Globus-M) and well-pronounced LH transitions with all the characteristic features of the transition were observed. The key indicators of the transition to H-mode include the reduction in D_α_, a positive break in the slope of plasma density, and periphery pressure gradients. 

**Figure 3:**
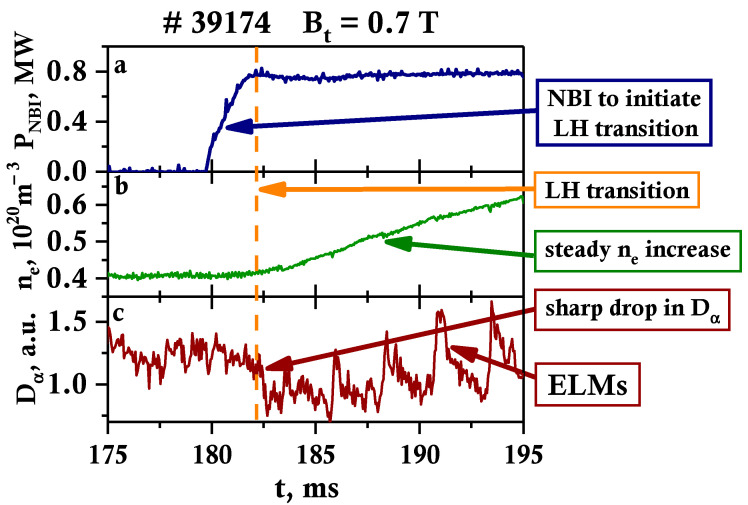
Temporal evolution of plasma parameters on Globus-M2 for discharge #39174: (**a**) NBI power P_NBI_, (**b**) electron density n_e_, and (**c**) D_α_ signal.

An example of an observed LH transition for discharge #39174 is presented in Figure 3. In this case, it was achieved with the following main parameters of the deuterium plasma: toroidal magnetic field B_t_ = 0.7 T; plasma current I_p_ = 280 kA; averaged electron density <n_e_> = (3–6) × 10^19^ m^−3^; electron temperature in the plasma core T_e_ = 0.6–1.1 keV; elongation *k* ≈ 1.9; triangularity δ ≤ 0.5. The direction of the magnetic field was chosen in such a manner that the toroidal ion drift was directed toward the X-point (lower null magnetic configuration).

For discharge #39174, the LH transition was initiated by the deuterium neutral beam injection (NBI) with particle energy 28 keV and heating power 0.8 MW (Figure 3a). The beam impact parameter is equal to 32 cm and the average pitch angle of the deposited fast ions is approximately 40°. The LH transition started at 182 ms, 2 ms after the neutral beam injection, which is indicated by the vertical orange line in Figure 3. On Globus-M2 deuterium, NBI was not the only method to achieve LH transition, as the transition to H-mode was also observed when using hydrogen NBI, as well as two NBI sources, were introduced into the plasma at different times. This method used does not dramatically affect the LH transition itself. The NBI allowed to effectively heat the plasma, as the electron and ion temperatures in the plasma core exceeded 1 keV.

During the LH transition, one may observe the expected increase in electron density n_e_ in Figure 3b and a drop in D_α_ in Figure 3c. The transition to the H-mode also leads to the appearance of edge localized modes (ELMs), which are a sign of the formation of a strong pressure gradient at the plasma edge [27]. They can be seen in the form of periodical D_α_ bursts (Figure 3c).

After the transition to H-mode, an increase in the plasma density was followed by the rise of the total stored energy W_DIA_, measured by the diamagnetic loop. Compared to the experiments on Globus-M, carried out with a magnetic field of 0.4 T, the total stored energy increased by a factor of four [10]. The h-factor also changes after the LH transition and increases from 0.5 to 1.2–1.4, which is the result of the energy confinement time τ_E_ in H-mode doubling and reaching 7 ms. While the NBI pulse is active, a quasi-steady phase state of the H-mode was established, characterized by a $\frac{dn}{dt}\;\approx \;0$ and $\frac{dW_{DIA}}{dt}\;\approx \;0$ for 10–15 ms, which is 1.5–2 times more than the τ_E_ for the discussed discharge.

### 3.1. Velocity Behavior during the LH Transition

The in-phase and quadrature (IQ) signals obtained using DBS were used for calculating the Doppler frequency shift, which corresponds to the poloidal rotation velocity of the plasma. The temporal evolution of the plasma rotation velocity during the LH transition was also investigated. 

**Figure 4:**
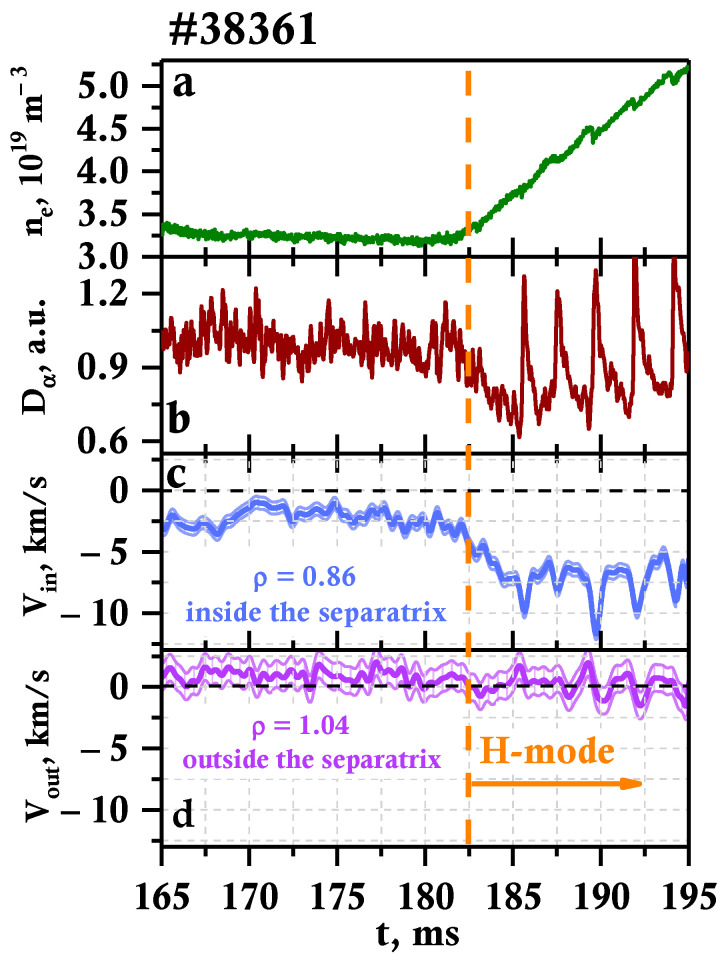
Temporal evolution of different plasma parameters on Globus-M2 during the LH transition: (**a**) electron density n_e_, (**b**) D_α_ signal, (**c**) plasma rotation velocity at ρ = 0.86, and (**d**) plasma rotation velocity at ρ = 1.04.

An example of the rotation velocity calculated for discharge #38361 by the “phase derivative” method smoothed over a time interval of 128 μs is presented in Figure 4. Two velocity evolutions are depicted: Figure 4c shows the behavior inside the separatrix at ρ = 0.86, while Figure 4d shows outside at ρ = 1.04. The vertical dashed line at 182.5 ms indicates the LH transition. One may observe that the poloidal rotation velocity inside the separatrix was around 3 km/s in the direction of the electron diamagnetic drift during the L-mode and increased rather dramatically during the transition to the H-mode, reaching the average value of 9 km/s. The behavior of the temporal evolution for plasma outside the separatrix differs. The velocity value does not exceed 2 km/s and is predominantly directed toward the ion diamagnetic drift pre-LH transition. Afterward, its value decreases closer to 0 km/s and its direction becomes harder to judge, which is highlighted by the error bars in Figure 4d. 

It is also worthy of note that in H-mode a periodic change in velocity values was observed. The comparison of the D_α_ signal (Figure 4b) and the velocity leads to the observation that there is a correlation between ELMs and the bursts of increasing velocity seen. The appearance of each ELM leads to a rapid increase in values of up to 11 km/s in Figure 4c, while during the inter-ELM periods, the velocity decreases once again; nonetheless, it remains around 7 km/s. In the case of Figure 4d, the direction of the rotation is also affected, with it changing during an ELM burst. Modeling of the reaction of the DBS signal to filaments was performed in [28], and the results highlighted that filaments that can develop during an ELM burst can impact the velocity values due to the backscattering being of a non-linear nature.

The radial profiles of the plasma rotation velocity are also worth investigating before and after the LH transition. It is possible to obtain these measurements using the DBS system described earlier. Due to the existence of multiple probing frequencies, the values are gathered simultaneously from different radii in the tokamak plasma. As has already been mentioned, the probing frequencies for these experiments were 48, 39, 29, and 20 GHz, with 48 GHz corresponding to the deeper part of the plasma and 20 GHz to the periphery of the plasma.

The radii are obtained by means of a ray tracing code, described in Section 2.1, as it is designed not only to calculate the probing beam trajectory but to also present information regarding the cut-off, such as the wave vector values and radii of the cut-off [26]. Two different moments in time were chosen to calculate the velocity profiles before and after the LH transition. The first profile was obtained for t_1_ = 180 ms during the L-mode, while the second one for t_2_ = 190 ms during H-mode. Table 1 contains the data of the calculated radii and corresponding wave vector values used for the profiles presented. One can observe a change in the cut-off radii associated with a change in electron density after the transition. The position of the cut-offs shifts toward the inner plasma region by approximately 1 cm, with the 48 GHz channel being the exception. Its radius value increases, meaning it moves slightly closer to the periphery.

**Figure 5:**
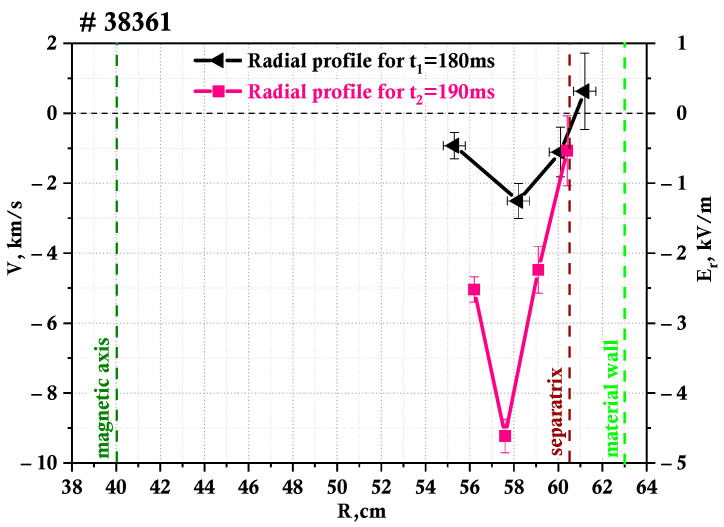
Plasma rotation velocity and electric field profile, measured by DBS.

The resulting profiles are presented in Figure 5. The figure shows the radial profile of both the rotation velocity and electric field, which were calculated using DBS measurements. The electric field can easily be calculated assuming that the velocity measured is indeed the E × B drift. Two different scales are used to indicate the obtained values: the one on the left for the rotation velocity, and the one on the right for the electric field. Additionally, different colors are used to indicate the different moments in time for which the profiles are calculated. The black line corresponds to 180 ms before the LH transition and the pink line to 190 ms after the LH transition. A clear peak is present on both profiles at around r = 58 cm. The absolute values of the velocity and electric field decrease closer to the periphery and the core. One can see a significant increase in the values for all radii after the LH transition, for example, in the case of the rotation velocity from 2.5 km/s to 9.2 km/s at its maximum. It is also worthy of note that for the 180 ms profile, there is a change in the sign of the velocity values at some radius, as at r = 61.2 cm the value is positive, and negative at r = 60.1 cm. This change in the direction of the rotation is not observable in the 190 ms case in the data available.

**Figure 6:**
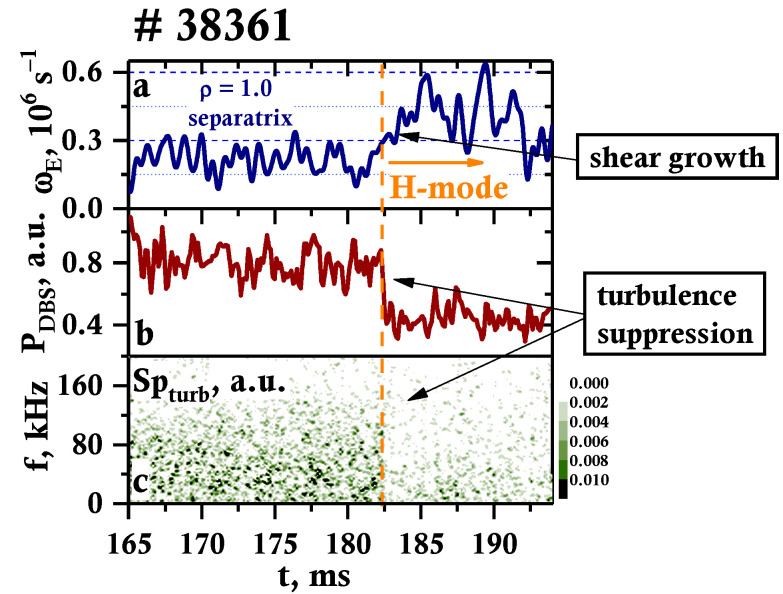
Temporal evolution of: (**a**) velocity shear, (**b**) DBS amplitude, and (**c**) DBS amplitude spectrogram.

The multi-frequency DBS system allows the following of the inhomogeneity of the poloidal rotation velocity, which is necessary for the determination of the rotation velocity shear. The analysis of the change in its behavior associated with the LH transition is of importance. Figure 6a demonstrates the temporal evolution of the rotation velocity shear for discharge #38361, calculated at the separatrix at ρ = 1.0. During L-mode, the value was around 3 × 10^−5^, which is believed to be the threshold value that initiates the transition to H-mode in Globus-M/M2. This effect was previously predicted in modeling works and a similar shear value was observed in other devices [29,30]. After the LH transition, there is a nearly two-fold increase in velocity shear. This is accompanied by a simultaneous decrease in the amplitude of the backscatter signal in Figure 6b, which is proportional to the amplitude of turbulence with wave number values in Table 1. This can also be observed in the form of a decrease in the intensity of all frequency components in the spectrogram of DBS amplitude in Figure 6c. This phenomenon is due to the fact that the shear plays a significant role in the suppression of anomalous transport and contributes to the plasma’s stable H-mode.

### 3.2. Turbulence Behavior during the LH Transition

**Figure 7:**
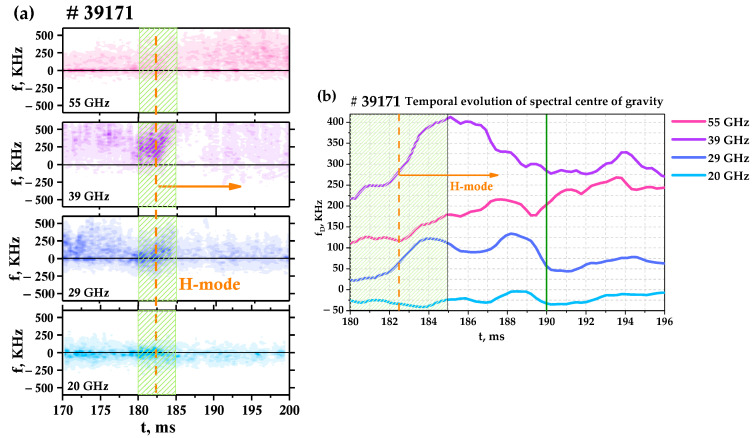
(**a**) Spectrograms of DBS complex signals for discharge #39171 and (**b**) temporal evolution of spectral center of gravity.

As was made evident in Figure 6, suppression of plasma turbulence is observed, so it is important to investigate the properties of turbulence for discharges with a transition to H-mode. For the purpose of investigating the core plasma during the LH transition, a 55 GHz frequency channel was also added to the DBS system, shown in Figure 2 [31]. This was conducted for the #39171 discharge, which is presented in Figure 7. The spectrograms of the complex DBS signals were calculated for the available frequencies of 20, 29, 39, and 55 GHz, and can be seen in Figure 7a. The intensity of each of these spectrograms is proportional to the turbulence amplitude, and the position of the center of gravity of the spectrum at each moment in time is proportional to the plasma rotation velocity [12]. This is why the temporal evolution of the spectral center of gravity was investigated as well, presented in Figure 7b. Different signs of the shift of the center of gravity correspond to the rotation of the plasma in opposite directions. 

The characteristics of discharge #39171 were similar to #38361 (see Figure 4, Figure 5 and Figure 6), however, the NBI power was reduced. This led to a slight change in the LH transition. The main differences were the plasma density being lower during the LH transition, and the D_α_ emission decreasing and the plasma density increasing slower during the transition to H-mode. However, the data collected regarding turbulence behavior remained consistent despite this. 

The LH transition is marked by the orange vertical line at 182.5 ms in Figure 7a. Right after the transition to H-mode (see the segment in the green box in Figure 7), there is a noticeable shift of the spectra to a higher-frequency region for the 29, 39, and 55 GHz channels (inner plasma region), while this is not observable for the 20 GHz channel (close to the separatrix). For example, in Figure 7b, it can be seen how frequency shift values increase dramatically from 250 to 410 kHz in a matter of 3 ms after the LH transition. Such a steady growth of the frequency shift values is also true for the 29 and 55 GHz probing frequencies during this time. However, after that, the frequency shift values decrease in the 29 and 39 GHz frequencies case. This can be explained by the fact that during this time, in the H-mode, the electron density continues to increase, leading to the movement of the cut-off position closer to the separatrix. This behavior of the center of gravity of the spectrograms corresponds to the rotation velocity behavior, which also grows after the LH transition, as shown in Figure 4c,d. It is also worthy of note that the spectral power decreases after a several-milliseconds delay (when a quasi-stationary H-mode is achieved after 185 ms), primarily on the peripheral channels. This can be seen as a decrease in color intensity in the spectrograms. The turbulence suppression is most pronounced for the 39, 29, and 20 GHz channels or in the 0.55–0.58 m radii interval. One can see that the spectral power does not change significantly for the 55 GHz channel.

**Figure 8:**
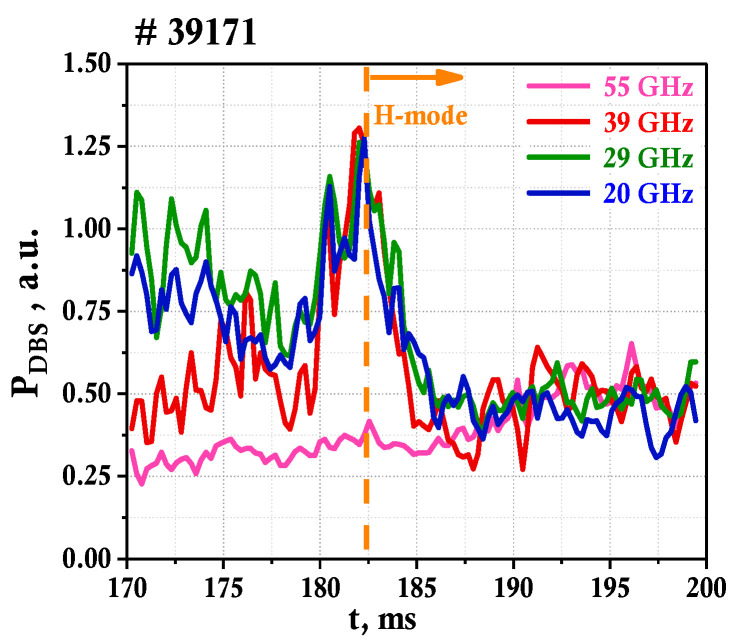
Temporal evolution of DBS power for discharge #39171.

These observations are confirmed by the obtained temporal evolution of the spectral power of the fluctuations that is presented in Figure 8. The different DBS probing frequencies are depicted using different colors as indicated in the figure. The LH transition is also marked using an orange vertical line at 182.5 ms. As the absolute power levels of the backscattered radiation were not measured and the sensitivity of the detectors differs for different probing frequencies, the results were obtained in the form of normalized power levels at t = 190 ms. The temporal evolution of each channel is presented in the corresponding calculated relative units. The change in fluctuation level is virtually identical for 39, 29, and 20 GHz, as it drops by almost half in the span of 3 ms right after the transition to H-mode. The 55 GHz channel exhibits dramatically different behavior with a slight increase in spectral power observed after the LH transition, meaning no turbulence suppression takes place in this area of the plasma. 

**Figure 9:**
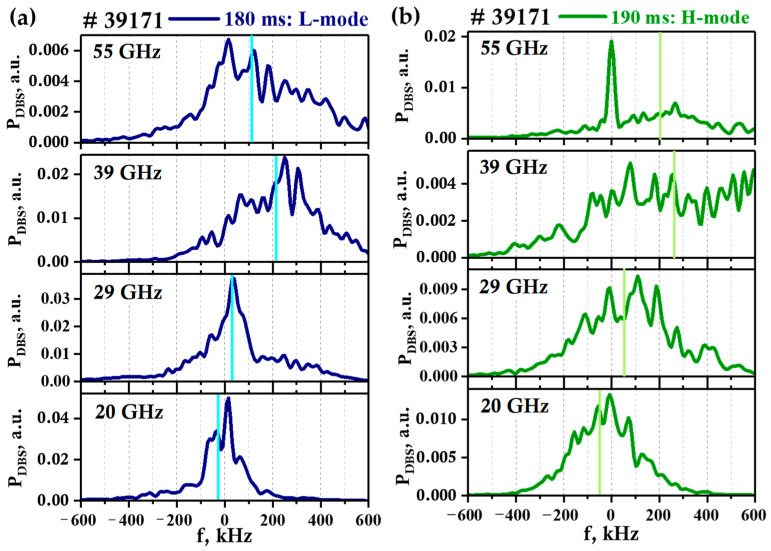
Spectra of DBS complex signals for discharge #39171 at: (**a**) 180 ms in L-mode; (**b**) 190 ms in H-mode.

Additionally, Figure 9 depicts the spectra of the DBS complex signals at two different moments in time. They were calculated at 180 ms before the transition to the H-mode, shown in Figure 9a, and at 190 ms after the transition when a quasi-stationary H-mode is achieved, shown in Figure 9b. Additionally, vertical lines that correspond to the center of gravity of the spectrum are introduced (see Figure 7b for comparison). The observable frequency shift of the spectra can be attributed to the rotation of the plasma. The positive frequency shift corresponds to the movement of the fluctuations in the electron diamagnetic drift direction. The largest shift is observed for the 39 GHz probing frequency and it even increases in H-mode. In addition, it can be easily determined that the Doppler shift at the 20 GHz frequency is not significant before and after the transition to the H-mode. It is also observable that there is a decrease in spectral power at 190 ms for the 39, 29, and 20 GHz channels. 

**Figure 10:**
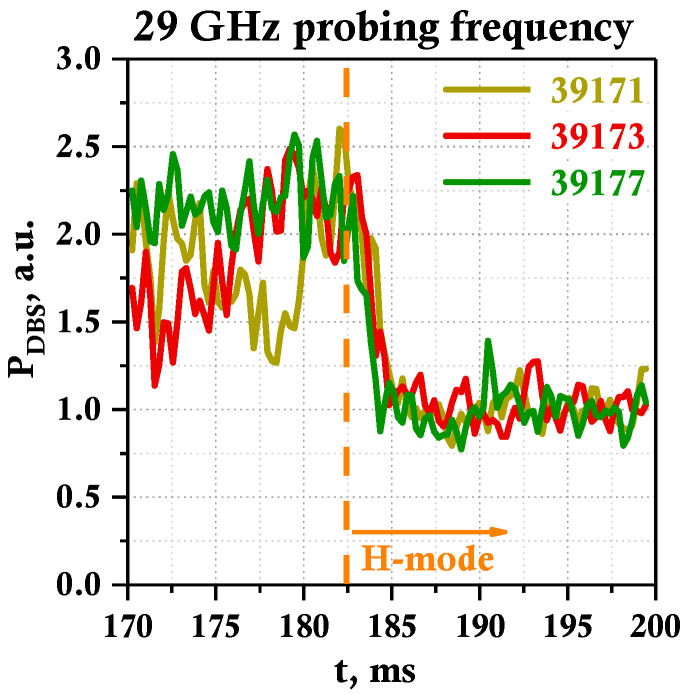
Temporal evolution of DBS power for 29 GHz probing frequency for discharges #39171, #39173, and #39177.

To highlight the repetitive nature of the observed turbulence suppression after the LH transition, the temporal evolution of the spectral power for several discharges was compared. The results are presented in Figure 10 for the probing frequency of 29 GHz (plasma periphery) for a series of discharges (#39171 shown in yellow, #39173 shown in red, and #39177 shown in green) with a transition to H-mode. The moment of the LH transition at around 182.5 ms is indicated by the orange vertical line. Here, a drop in the turbulence level is observed in all discharges and it is almost identical in behavior and values. It can be noted that the relative level decreases by about a factor of 2.

## 4. Summary

The Doppler backscattering diagnostic was installed on the upgraded spherical tokamak Globus-M2 and has been successfully used since the beginning of the experimental studies on the device. Its description, as well as an example of its implementation are presented and discussed. The use of DBS for the study of the LH transition is demonstrated. DBS measurements of the poloidal plasma velocity and small plasma turbulence allowed for it to be investigated by observing the behavior of these parameters. 

First, measurement results of the poloidal rotation velocity profile using a multi-frequency DBS system on Globus-M2 are presented in the work. In experiments, for ρ = 0.7–1.0, the plasma was observed to rotate in the direction of electron diamagnetic drift, while outside the separatrix in the direction of the ion diamagnetic drift. In the pedestal region, the absolute value of the velocity was seen to increase significantly during the LH transition from 3 km/s in L-mode to 9 km/s in H-mode. The velocity at the separatrix did not change much after the transition and remained close to zero. The multi-frequency probing approach of the DBS method allowed investigation of the spatial inhomogeneity of the rotation velocity and, thus, the calculation of the velocity shear, which is an important characteristic associated with the LH transition. The velocity shear value was found to be close to 3 × 10^−5^, right before the transition to H-mode. This value is believed to be the threshold value for the LH transition on the Globus-M2 tokamak. 

Additionally, the plasma density fluctuations with wave vector values of 2–7 cm^−1^ have been studied on Globus-M2 using DBS. During the LH transition, the suppression of such small-scale plasma turbulences was observed in the temporal evolution of the spectral power of the DBS signals. The information obtained about the turbulence amplitude in different discharge areas indicates that the turbulence suppression is of a peripheral nature. These results were compared for a series of discharges, and the repetitive nature of the turbulence suppression in the periphery after the LH transition was also noted.

The value of the measured velocity shear being close to the threshold value, together with the rapid decrease in the level of peripheral turbulence, fits well into the concept of the transition to H-mode by shear turbulence suppression.

## Figures and Tables

**Table 1:** Radii and wave vector values for discharge #38361 calculated by a ray tracing code.

#38361	180 ms (before the LH Transition)		190 ms (after the LH Transition)	
f, GHz	R, cm	$k_{⏊}$, cm^−1^	R, cm	$k_{⏊}$, cm^−1^
20	61.2	2.2	60.4	2.4
29	60.1	3.4	59.1	3.6
39	58.2	4.8	57.6	5.0
48	55.3	6.4	56.2	6.6

## Data Availability

Not applicable.
